# Moderate intra-abdominal hypertension is associated with an increased lactate-pyruvate ratio in the rectus abdominis muscle tissue: a pilot study during laparoscopic surgery

**DOI:** 10.1186/2110-5820-2-S1-S14

**Published:** 2012-07-05

**Authors:** Liivi Maddison, Juri Karjagin, Jyrki Tenhunen, Joel Starkopf

**Affiliations:** 1 Department of Anaesthesiology and Intensive Care, University of Tartu, Puusepa 8, Tartu, 51014, Estonia; 2 Anaesthesiology and Intensive Care Clinic, Tartu University Hospital, Puusepa 8, Tartu, 51014, Estonia; 3 Critical Care Medicine Research Group, Department of Intensive Care Medicine, Tampere University Hospital, Teiskontie 35, PL 2000, Tampere, 33521, Finland

**Keywords:** microdialysis, intra-abdominal pressure, intra-abdominal hypertension, lactate-to-pyruvate ratio, muscle ischemia, early clinical sign

## Abstract

**Background:**

The development of intra-abdominal hypertension [IAH] in critically ill patients admitted to the ICU is an independent predictor of mortality. In an attempt to find an early, clinically relevant metabolic signal of modest IAH, we investigated abdominal wall metabolite concentrations in a small group of patients undergoing laparoscopic surgery. We hypothesized that elevated intra-abdominal pressure [IAP] due to pneumoperitoneum leads to an increased lactate/pyruvate [L/P] ratio *in the rectus abdominis *muscle [RAM], indicating anaerobic metabolism.

**Method:**

Six patients scheduled for elective laparoscopic gastric fundoplication were studied. Two hours before surgery, a microdialysis catheter (CMA 60, CMA Small Systems AB, Solna, Sweden) was inserted into the RAM under local anaesthesia. Catheter placement was confirmed by ultrasound. The microdialysis perfusion rate was set at 0.3 μL/min. Dialysate was collected hourly prior to pneumoperitoneum, during pneumoperitoneum, and for 2 h after pneumoperitoneum resolution. IAP was maintained at 12 to 13 mmHg during the surgery. The glucose, glycerol, pyruvate and lactate contents of the dialysate were measured.

**Results:**

The median (interquartile range) L/P ratio was 10.3 (7.1 to 15.5) mmol/L at baseline. One hour of pneumoperitoneum increased the L/P ratio to 16.0 (13.6 to 35.3) mmol/L (*p *= 0.03). The median pneumoperitoneum duration was 86 (77 to 111) min. The L/P ratio at 2 h post-pneumoperitoneum was not different from that at baseline (*p *= 1.0). No changes in glycerol or glucose levels were observed.

**Conclusions:**

IAH of 12 to 13 mmHg, even for a relatively short duration, is associated with metabolic changes in the abdominal wall muscle tissue of patients undergoing laparoscopic surgery. We suggest that tissue hypoperfusion occurs even during a modest increase in IAP, and intramuscular metabolic monitoring could therefore serve as an early warning sign of deteriorating tissue perfusion.

## Introduction

The development of intra-abdominal hypertension [IAH] in critically ill patients admitted to the ICU is an independent predictor of mortality [1]. The incidence of IAH is as high as 20% to 40%, while the most severe form of IAH, abdominal compartment syndrome [ACS], occurs in 5% to 10% of ICU patients [2-6].

The diagnosis of IAH/ACS is made based on the measurement of intra-abdominal pressure [IAP], and it is dependent on the accuracy and frequency of the measurements [7]. To date, no clinically relevant biochemical markers have been available to indicate when IAH/ACS becomes clinically significant and thereby begins to affect end-organ perfusion. The lower threshold for the diagnosis of IAH is arbitrarily defined and supported by only a few epidemiological studies [2,8]. Although IAP exceeding 12 mmHg is considered a mild form of IAH, it does not usually lead to an immediate change in clinical behaviour or decision-making [3]. At IAP values ranging from 12 to 15 mmHg, many clinicians simply wait and monitor the progression of the clinical condition rather than make changes to the treatment. Decreasing urine output is often the primary clinical trigger for initiating treatment to decrease IAP. However, it is reasonable to assume that tissue perfusion may have already been severely jeopardized by the time of onset of oliguria or anuria. In the present pilot study, we examined whether a short-lasting IAH at 12 mmHg is associated with altered oxidative tissue metabolism indicative of tissue hypoperfusion.

Microdialysis has been successfully used in animal experiments to assess metabolic changes during IAH. A high lactate-pyruvate [L/P] ratio was detected in the dialysate from the rectus abdominis muscle [RAM] in animal models of severe IAH [9]. More generally, microdialysis has been used to monitor oxidative metabolism in patients with severe brain injury as well as during liver transplantation, plastic surgery and cardiovascular surgery [10-13]. In all of these studies, an increased L/P ratio has been used as a marker of tissue ischemia.

In an attempt to find an early metabolic signal of IAH, we measured the abdominal wall metabolite concentrations in a small group of patients undergoing laparoscopic surgery. We hypothesized that IAP elevation during pneumoperitoneum leads to insufficient tissue perfusion of the RAM and that the L/P ratio is increased in parallel as a signal of anaerobic metabolism.

## Materials and methods

The study was approved by the University of Tartu Ethics Review Committee on Human Research (protocol number: 170/T-11 28.04.2008). Informed consent was obtained prior to inclusion in the study. The study was performed in accordance with the Helsinki Declaration. Because this was a prospective observational study and not an interventional trial, we did not register the study with clinicaltrials.gov.

### Patients

Eight patients scheduled for elective laparoscopic surgery were enrolled. Two of these patients were excluded because of catheter misplacement during the surgery. All patients underwent laparoscopic gastric fundoplication. Altogether, three women and three men with a median age of 36 (30 to 42) years were studied. The patients' median body mass index was 26.2 (23.3 to 28.1), and the median haemoglobin concentration before surgery was 145 (137 to 160) g/L.

### Anaesthesia and surgery

Five patients received sodium thiopental and one patient received propofol for the induction of general anaesthesia. During all surgeries, anaesthesia was maintained with sevoflurane with FiO_2 _ranging from 30% to 50%, as decided by the attending anaesthesiologist. Atracurium besylate (GlaxoSmithKline, Greenford, UK) was used as a muscle relaxant. To ensure an equal degree of muscle relaxation in every patient, the dose was adjusted according to kinesiomyography (E-NMT, Datex, Helsinki, Finland). Ventilators were set in volume-controlled mode and regulated to achieve normal (35 to 45 mmHg) end-tidal CO_2_. The end-expiratory pressure was set at 0 cmH_2_O. Patient vital signs during anaesthesia were recorded with a GE Aisys anaesthesia machine (GE Datex Ohmeda Aisys, Helsinki, Finland). Blood samples for lactate measurements were drawn from an arterial cannula before and after pneumoperitoneum. Pneumoperitoneum was introduced by insufflation of CO_2 _at the beginning of surgery. The IAP was held at 12 to 13 mmHg using an automated insufflator.

### Microdialysis

Two hours before surgery, a microdialysis catheter (CMA 60, CMA Small Systems AB, Solna, Sweden) was inserted into the RAM under local anaesthesia (2% lidocaine). Catheter placement was confirmed by ultrasound (MicroMaxx, SonoSite Inc., Bothell, WA, USA). The microdialysate perfusion rate was set at 0.3 μL/min. Dialysate was collected hourly prior to pneumoperitoneum, during pneumoperitoneum, and for 2 h after pneumoperitoneum resolution. The samples were stored in a freezer at -80°C for a maximum of 10 months at Tartu University Hospital, Estonia and were sent to Tampere University Hospital in a single shipment for further analysis. The glucose, lactate, pyruvate and glycerol contents of the microdialysates were measured with a CMA 600 analyser (CMA Small Systems AB, Solna, Sweden).

### Statistical analyses

This was a pilot study, and prior RAM L/P ratio data for IAH in humans were not available. We were therefore not able to perform any meaningful power calculations for sample size estimation. Rather, these data will be used for those purposes in future studies to allow for adequate sample size calculation. The first analysis was undertaken after obtaining data from six patients. Statistical analysis was performed using GraphPad Prism 5.02 (GraphPad Software, Inc., San Diego, CA, USA). The Wilcoxon matched-pairs test (non-parametric) was used to test the median differences in paired data. Data are presented as medians with interquartile ranges. Differences were considered statistically significant at *p *< 0.05.

## Results

The surgery and clinical course were uneventful in each patient. One patient experienced syncope before anaesthesia during intravenous line insertion. This resolved quickly upon the intravenous [IV] injection of 1 mg atropine. During the surgery, this patient's mean arterial pressure [MAP] was 94 (88 to 103) mmHg. The average volume of IV infusions administered during the operations was 1,600 (1,375 to 1,825) mL. The median pneumoperitoneum duration was 86 (77 to 111) min. IAP during the surgery was stable at 12 (12 to 13) mmHg. The patient's global haemodynamics were stable. The median MAP during pneumoperitoneum was 77 (74 to 94) mmHg, and the median abdominal perfusion pressure [APP] was 65 (62 to 82) mmHg. The median duration of MAP ≤ 65 mmHg was 1.5 (0.0 to 13.8) min. The median blood lactate level was 0.7 (0.6 to 1.4) mmol/L before and 1.0 (0.6 to 1.5) mmol/L after pneumoperitoneum (*p *= 0.2). The median arterial pCO_2 _was 34.6 (32.9 to 39.9) mmHg before and 43.0 (42.0 to 45.7) mmHg after pneumoperitoneum (*p *= 0.003).

**Figure 1:**
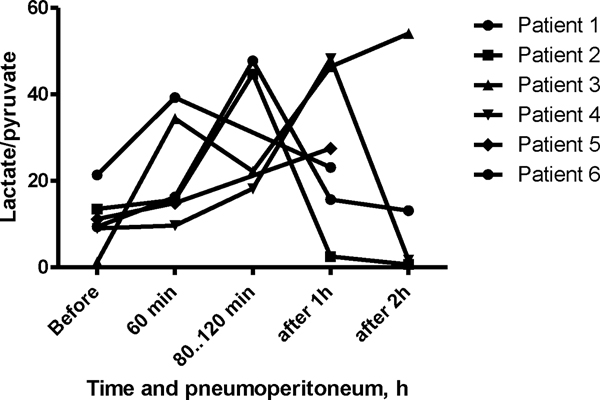
**Individual L/P ratios in the RAM during laparoscopic surgery**.

**Figure 2:**
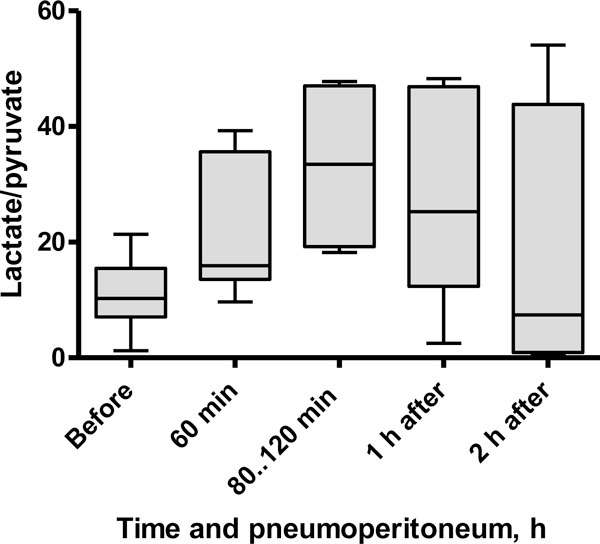
**Box plot showing the median L/P ratio in the RAM during laparoscopic surgery**.

The L/P ratio increased during pneumoperitoneum in all patients. The median L/P ratio was 10.3 (7.1 to 15.5) mmol/L at baseline, and it increased to 16.0 (13.6 to 35.6) mmol/L after 1 h of pneumoperitoneum (*p *= 0.03). L/P ratio values were not correlated with MAP or APP. Individual dynamics are shown in Figure 1. The patient who suffered from syncope at preparation to anaesthesia had normal baseline values, but a marked increase was observed in the L/P ratio, from 1.2 to 54.1 mmol/L, during pneumoperitoneum. This individual maintained a high L/P ratio during the 2 h of observation after pneumoperitoneum. In the other patients, we observed a gradual decrease of the L/P ratio to the previous baseline level by 2 h after the end of pneumoperitoneum (Figure 2).

**Figure 3:**
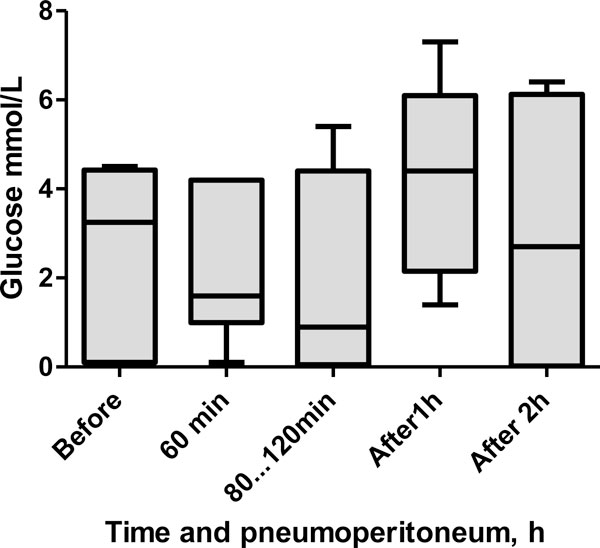
**Box plot showing median glucose levels in the interstitium of the RAM during laparoscopic surgery**.

The median RAM tissue glucose level at baseline was 3.3 (0.1 to 4.4) mmol/L (Figure 3). The surgery did not cause significant changes, although a trend of decreasing glucose levels during pneumoperitoneum was observed. At 60 min of pneumoperitoneum, the glucose level was 1.6 (1.0 to 4.2) mmol/L (*p *= 0.44), and at 80 to 120 min, the level was 0.9 (0.1 to 4.4) mmol/L (*p *= 0.81). The blood glucose level increased to 4.4 (2.1 to 6.1) mmol/L (*p *= 0.06) at 1 h after pneumoperitoneum and was 2.7 (0.02 to 6.1) mmol/L (*p *= 0.75) at 2 h after the pneumoperitoneum.

**Figure 4:**
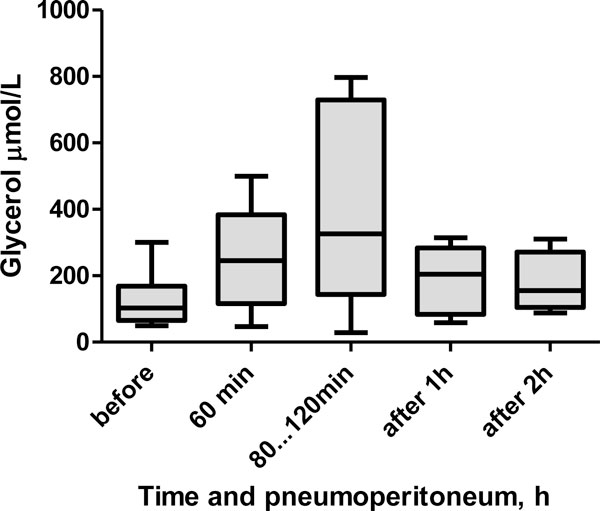
**Box plot showing median glycerol levels in the interstitium of the RAM during laparoscopic surgery**.

The median glycerol level at baseline was 103 (65 to 169) μmol/L (Figure 4). During pneumoperitoneum, the level increased to 245 (117 to 384) μmol/L (*p *= 0.06) at 60 min and to 326 (144 to 730) μmol/L (*p *= 0.13) at 80 to 120 min. At 1 h after pneumoperitoneum, the glycerol level was 204 (84 to 284) μmol/L (*p *= 0.31), and at 2 h after pneumoperitoneum, the glycerol level was 155 (105 to 272) μmol/L (*p *= 0.63).

## Discussion

The present study demonstrates that IAP increases to only 12 mmHg for a relatively short duration are associated with metabolic changes in the abdominal wall muscle tissue of patients undergoing laparoscopic surgery. This indicates that anaerobic metabolism, and thereby RAM hypoperfusion, occurs upon very modest increases of IAP.

Several recent studies have shown that an increase in the mean IAP is associated with adverse ICU outcomes [2,14-16]. However, the time course of IAP-related adverse events in humans is poorly understood. To address this issue, we used microdialysis-aided sample collection from the extravascular space of the RAM. The RAM is surrounded by a tight sheet of fascia, which makes the muscle-fascia compartment relatively noncompliant. Thereby, the pressure in the intra-abdominal cavity is reflected to the muscle tissue and its perfusion. Meier and co-workers used a similar microdialysis approach in a rat model of IAH. They showed that during a 3-h period of IAP at 20 mmHg, the L/P ratio in the RAM tissue increased significantly, indicating ischemia and energy failure. To the best of our knowledge, the present study is the first to make similar observations in humans. Our findings are generally in accordance with previous animal experiments [9]. Notably, metabolic changes in the human RAM tissue were observed at lower levels of IAP than those that were found to cause metabolic changes in rats.

The limitations of our study include its small sample size and microdialysis-related problems, specifically catheter displacement before or during surgery in two patients. The first limitation is expected, given that we are reporting these results as a pilot trial. The latter reflects the limitations of the method *per se*. Microdialysis is an invasive, relatively expensive and time-consuming procedure. Therefore, this study lacks comparative data from control patients or other anatomical regions of the same patient. Because the insertion of a central venous line was not clinically indicated in these patients, we also did not measure global indices of preload and circulation, which would be required for an in-depth interpretation of the microdialysis data. However, the observed arterial blood pressure and heart rate dynamics do not suggest remarkable alterations in cardiac output during pneumoperitoneum.

The L/P ratio of a tissue reflects its reaction to changing oxygen and glucose supplies [17], and it is a better marker of cell ischemia than lactate alone [18]. Lactate might also be produced under aerobic conditions [19], while the L/P ratio is a specific marker of anaerobic conditions. In severe sepsis, the L/P ratio is an independent predictor of 28-day mortality [20]. One possible explanation for this observation is that it may reflect the reduced activity of pyruvate dehydrogenase, which leads to the anaerobic metabolism of pyruvate to lactate and an elevated L/P ratio [19,21]. During endotoxin shock, the L/P ratio of pre-hepatic venous blood can be increased due to the intestinal uptake of pyruvate [22].

During ischemia, both the oxygen and the glucose supply to the tissue are compromised. Inadequate delivery combined with increased uptake leads to a marked decrease in the interstitial glucose levels during severe ischemia [23]. We observed a decrease in dialysate glucose levels in our patients, although this trend was not statistically significant. It may be speculated that a moderate increase in IAP due to pneumoperitoneum for laparoscopy is not severe enough to deprive glucose delivery to the RAM tissue in these patients.

Elevated tissue glycerol levels indicate cell membrane damage. Bäckström et al. have demonstrated that mesenteric vein glycerol is an indicator of splanchnic ischemia [19]. We observed no significant changes in RAM glycerol; however, there was a trend toward higher glycerol levels during pneumoperitoneum, which may further support the idea that tissue injury is caused by increased IAP.

IAH has a global impact on the human body. It results in a variable series of pathophysiological consequences, the specifics of which depend on the underlying diagnosis [24]. Although epidemiological studies suggest a threshold of 12 mmHg for the diagnosis of IAH, it is doubtful whether a single IAP level can be universally applied as a critical level for all patients. It remains unclear whether any subclinical effects of IAP may be inferred before detectable organ dysfunction. One good example of a work on this topic is a study by Kirkpatrick et al., who demonstrated that ultrasound measurements of renal blood flow correlate with IAH in pigs [25]. Renal blood flow was impaired prior to a decrease in urine output, an obvious and common symptom of renal dysfunction caused by increased IAP. These results are supported by those of Wauters et al., who showed that IAH is associated with decreased renal blood flow and blood flow redistribution away from the kidney [26]. Olofsson et al. have demonstrated altered intestinal microcirculation in a similar experimental setting with a stepwise increase in IAP by CO_2 _insufflation [27]. In all of these animal experiments, significant changes were described at markedly high levels of IAP. The results of the present study, in which an increased L/P ratio in the rectus muscle tissue of our patients was observed after only 1 h of moderate IAH, are surprising. However, it remains unclear whether the observed metabolic changes in RAM are directly related to the altered perfusion of intra-abdominal organs, whether they are related to clinical outcome and whether patient treatment should be modified based on these metabolic changes. The results indicate that IAP levels of 12 mmHg are associated with unfavourable metabolic conditions, and they therefore support the recommended IAH grading [5]. The European practice guidelines for pneumoperitoneum in laparoscopic surgery states that IAP levels higher than 12 mmHg should be avoided and that the duration of the procedure must be kept as short as possible [28].

## Conclusions

The present study demonstrates that even a mild to moderate increase of IAP for a short duration is associated with unfavourable metabolic changes in the RAM. Further investigation is required to evaluate whether microdialysis can be used as a diagnostic tool for the detection and grading of IAH.

## Abbreviations

ACS: abdominal compartment syndrome; APP: abdominal perfusion pressure; GE: General Electric; IAH: intra-abdominal hypertension; IAP: intra-abdominal pressure; IV: intravenous; L/P ratio: lactate-to-pyruvate ratio; MAP: mean arterial pressure; RAM: rectus abdominis muscle.

## Competing interests

The authors declare that they have no competing interests.

## Authors' contributions

LM participated in the design of the study, data acquisition and analysis, and drafted the first version of the manuscript. JK was involved in the study design and data acquisition, and revised the manuscript. JS contributed to the concept and the design of the study, and revised the manuscript. JT contributed to the laboratory analysis, data processing and critical revision of the manuscript. All authors have read and approved the final manuscript.

## References

[B1] Malbrain ML Deeren D De Potter T Intra-abdominal hypertension in the critically ill: it is time to pay attention Curr Opin Crit Care 2005 11 156 157 10.1097/01.ccx.0000155355.86241.1b 15758597

[B2] Malbrain ML Chiumello D Pelosi P Bihari D Innes R Ranieri VM Del Turco M Wilmer A Brienza N Malcangi V Cohen J Japiassu A De Keulenaer BL Daelemans R Jacquet L Laterre PF Frank G de Souza P Cesana B Gattinoni L Incidence and prognosis of intraabdominal hypertension in a mixed population of critically ill patients: a multiple-center epidemiological study Crit Care Med 2005 33 315 322 10.1097/01.CCM.0000153408.09806.1B 15699833

[B3] Vidal MG Weisser JR Gonzalez F Toro MA Loudet C Balasini C Canales H Reina R Estenssoro E Incidence and clinical effects of intra-abdominal hypertension in critically ill patients Crit Care Med 2008 36 1823 1831 10.1097/CCM.0b013e31817c7a4d 18520642

[B4] Ball CG Kirkpatrick AW McBeth P The secondary abdominal compartment syndrome: not just another post-traumatic complication Can J Surg 2008 51 399 405 18841232 PMC2556543

[B5] Malbrain ML Cheatham ML Kirkpatrick A Sugrue M Parr M De Waele J Balogh Z Leppäniemi A Olvera C Ivatury R D'Amours S Wendon J Hillman K Johansson K Kolkman K Wilmer A Results from the International Conference of Experts on Intra-abdominal Hypertension and Abdominal Compartment Syndrome. I. Definitions Intensive Care Med 2006 32 1722 1732 10.1007/s00134-006-0349-5 16967294

[B6] De Waele JJ Hoste E Malbrain ML Decompressive laparotomy for abdominal compartment syndrome-a critical analysis Crit Care 2006 10 R51 10.1186/cc4870 16569255 PMC1550894

[B7] Cheatham ML Malbrain ML Kirkpatrick A Sugrue M Parr M De Waele J Balogh Z Leppäniemi A Olvera C Ivatury R D'Amours S Wendon J Hillman K Wilmer A Results from the International Conference of Experts on Intra-abdominal Hypertension and Abdominal Compartment Syndrome. II. Recommendations Intensive Care Med 2007 33 951 962 10.1007/s00134-007-0592-4 17377769

[B8] De Keulenaer BL De Waele JJ Powell B Malbrain ML What is normal intra-abdominal pressure and how is it affected by positioning, body mass and positive end-expiratory pressure? Intensive Care Med 2009 35 969 976 10.1007/s00134-009-1445-0 19242675

[B9] Meier C Contaldo C Schramm R Holstein JH Hamacher J Amon M Wanner GA Trentz O Menger MD Microdialysis of the rectus abdominis muscle for early detection of impending abdominal compartment syndrome Intensive Care Med 2007 33 1434 1443 10.1007/s00134-007-0725-9 17576536

[B10] Engstrom M Polito A Reinstrup P Romner B Ryding E Ungerstedt U Nordstrom CH Intracerebral microdialysis in severe brain trauma: the importance of catheter location J Neurosurg 2005 102 460 469 10.3171/jns.2005.102.3.0460 15796380

[B11] Nowak G Ungerstedt J Wernerson A Ungerstedt U Ericzon BG Hepatic cell membrane damage during cold preservation sensitizes liver grafts to rewarming injury J Hepatobiliary Pancreat Surg 2003 10 200 205 10.1007/s00534-002-0760-4 14605976

[B12] Roidmark J Heden P Ungerstedt U Prediction of border necrosis in skin flaps of pigs with microdialysis J Reconstr Microsurg 2000 16 129 134 10.1055/s-2000-7547 10706203

[B13] Langemann H Habicht J Mendelowitch A Kanner A Alessandri B Landolt H Gratzl O Microdialytic monitoring during a cardiovascular operation Acta Neurochirurg (Suppl) 1996 67 70 74 10.1007/978-3-7091-6894-3_16 8870807

[B14] Malbrain ML Chiumello D Pelosi P Wilmer A Brienza N Malcangi V Bihari D Innes R Cohen J Singer P Japiassu A Kurtop E De Keulenaer BL Daelemans R Del Turco M Cosimini P Ranieri M Jacquet L Laterre PF Gattinoni L Prevalence of intra-abdominal hypertension in critically ill patients: a multicentre epidemiological study Intensive Care Med 2004 30 822 829 10.1007/s00134-004-2169-9 14758472

[B15] Reintam A Parm P Kitus R Kern H Starkopf J Primary and secondary intra-abdominal hypertension-different impact on ICU outcome Intensive Care Med 2008 34 1624 1631 10.1007/s00134-008-1134-4 18446319

[B16] Reintam Blaser A Parm P Kitus R Starkopf J Risk factors for intra-abdominal hypertension in mechanically ventilated patients Acta Anaesthesiol Scand 2011 55 607 614 10.1111/j.1399-6576.2011.02415.x 21418151

[B17] Nowak G Ungerstedt J Wernerman J Ungerstedt U Ericzon BG Clinical experience in continuous graft monitoring with microdialysis early after liver transplantation Br J Surg 2002 89 1169 1175 10.1046/j.1365-2168.2002.02187.x 12190684

[B18] Birke-Sorensen H Andersen NT Metabolic markers obtained by microdialysis can detect secondary intestinal ischemia: an experimental study of ischemia in porcine intestinal segments World J Surg 2010 34 923 932 10.1007/s00268-010-0502-8 20195605

[B19] Bäckström T Liska J Oldner A Lockowandt U Franco-Cereceda A Splanchnic metabolism during gut ischemia and short-term endotoxin and hemorrhagic shock as evaluated by intravasal microdialysis Shock 2004 21 572 578 10.1097/01.shk.0000127069.65490.65 15167688

[B20] Kopterides P Nikitas N Vassiliadi D Orfanos SE Theodorakopoulou M Ilias I Boutati E Dimitriadis G Maratou E Diamantakis A Armaganidis A Ungerstedt U Dimopoulou I Microdialysis-assessed interstitium alterations during sepsis: relationship to stage, infection, and pathogen Intensive Care Med 2011 37 1756 1764 10.1007/s00134-011-2336-8 21847648

[B21] Gore DC Jahoor F Hibbert JM DeMaria EJ Lactic acidosis during sepsis is related to increased pyruvate production, not deficits in tissue oxygen availability Ann Surg 1996 224 97 102 10.1097/00000658-199607000-00015 8678625 PMC1235253

[B22] Tenhunen JJ Uusaro A Kärjä V Oksala N Jakob SM Ruokonen E Apparent heterogeneity of regional blood flow and metabolic changes within splanchnic tissues during experimental endotoxin shock Anesth Analg 2003 97 555 563 10.1213/01.ANE.0000072703.37396.93 12873953

[B23] Ohashi H Kawasaki N Fujitani S Kobayashi K Ohashi M Hosoyama A Wada T Taira Y Utility of microdialysis to detect the lactate/pyruvate ratio in subcutaneous tissue for the reliable monitoring of haemorrhagic shock J Smooth Muscle Res 2009 45 269 278 10.1540/jsmr.45.269 20093795

[B24] Sugrue ME Bukhari Y Intra-abdominal pressure and abdominal compartment syndrome in acute general surgery World J Surg 2009 33 1123 1127 10.1007/s00268-009-0040-4 19404708

[B25] Kirkpatrick AW Colistro R Laupland KB Fox DL Konkin DE Kock V Mayo JR Nicolaou S Renal arterial resistive index response to intraabdominal hypertension in a porcine model Crit Care Med 2007 35 207 213 10.1097/01.CCM.0000249824.48222.B7 17080005

[B26] Wauters J Claus P Brosens N McLaughlin M Malbrain M Wilmer A Pathophysiology of renal hemodynamics and renal cortical microcirculation in a porcine model of elevated intra-abdominal pressure J Trauma 2009 66 713 719 10.1097/TA.0b013e31817c5594 19276743

[B27] Olofsson PH Berg S Ahn HC Brudin LH Vikström T Johansson KJM Gastrointestinal microcirculation and cardiopulmonary function during experimentally increased intra-abdominal pressure Crit Care Med 2009 37 230 239 10.1097/CCM.0b013e318192ff51 19050608

[B28] Neudecker J Sauerland S Neugebauer E Bergamaschi R Bonjer HJ Cuschieri A Fuchs KH Jacobi Ch Jansen FW Koivusalo AM McMahon MJ Millat B Schwenk W The European Association for Endoscopic Surgery clinical practice guideline on the pneumoperitoneum for laparoscopic surgery Surg Endosc 2002 16 1121 1143 10.1007/s00464-001-9166-7 12015619

